# A Temporospatial Study of Sympathetic Skin Response and Electroencephalogram in Oral Mucosa Thermal Perception

**DOI:** 10.3389/fnins.2022.907658

**Published:** 2022-07-15

**Authors:** Hao Zhang, Shengjing Hu, Zhangang Wang, Xiang Li, Suogang Wang, Gang Chen

**Affiliations:** ^1^Department of Oral and Maxillofacial Surgery, School of Stomatology, Tianjin Medical University, Tianjin, China; ^2^School of Biomedical Engineering and Technology, Tianjin Medical University, Tianjin, China; ^3^Changzhou Hospital of Traditional Chinese Medicine, Changzhou, China; ^4^Department of Oral Surgery, School of Dentistry, University of Birmingham, Birmingham, United Kingdom

**Keywords:** sympathetic skin response, electroencephalogram, autonomic nervous system, oral mucosa, thermal perception

## Abstract

**Objective:**

To investigate the temporospatial changes in sympathetic skin response (SSR) and electroencephalogram (EEG) under thermal stimuli and to draw a topographic map of SSR threshold temperature of the oral mucosa.

**Materials and Methods:**

A total of 40 healthy volunteers (24 men, 16 women, mean age of 23 ± 3) were enrolled. Thermal stimuli were applied to the 35 partitions of oral mucosa starting from 36°C at the gradience of 1°C and the lowest temperature evoked SSR was defined as SSR threshold temperature. SSR and EEG signals at 45, 48, 51, and 54°C were then recorded synchronously.

**Results:**

The SSR threshold temperature increased from the anterior areas to the posterior areas. No significant difference between bilateral corresponding areas or between genders was observed. The SSR amplitude value increased from 45 to 54°C in the same area, while the highest value was recorded on the tip of the tongue and decreased backwardly from the anterior area. There were significant differences in latency of SSR between the tip of the tongue and the molar areas of the oral cavity (*p* < 0.05). Reduction in the alpha frequency band was observed after thermal stimuli, and there were statistical differences between baseline and thermal stimuli in all four degrees of temperatures (*p* < 0.05).

**Conclusion:**

The result of the experiment revealed that the autonomic and central nervous system (CNS) played important roles in thermal perception of oral mucosa and could be helpful for better understanding of pathological mechanism of burning mouth syndrome (BMS).

## Introduction

Thermal perception is one of the essential senses. As the initial part of the digestion system, oral mucosa played a significant role in perception, protection, and secretion by acting as a major barrier (Liu et al., 2015). Thermal stimuli that exceed the threshold of thermal perception of the oral mucosa can lead to unpleasant feelings and even injury (Petrus et al., 2007; Wei et al., 2011; Nakamura et al., 2012). In addition, in our clinical practice, the patient who suffered from burning mouth syndrome (BMS) usually complained about painful heat feeling without real stimuli and obvious mucosal pathology. This could be potentially explained by hypothesis of nerve damage or sensory abnormalities (Forssell et al., 2002; Hagelberg et al., 2003).

The sympathetic skin response (SSR) was referred to as the process of a transient change in skin action potential upon receipt of external or internal stimulation (Vetrugno et al., 2003). Hilz et al. (1999) demonstrated the feasibility of evoking SSR by means of thermal stimuli. Moreover, temperature nociceptors and receptors were involved in the nervous control of sweat glands (Karl et al., 1975). In addition, Koszewicz et al. (2012) revealed significant prolongation of SSR latency in the foot of patients with burning mouth syndrome. However, there were few studies on the difference in the temperature-sensing ability between various areas of the oral mucosa, and studies on the SSR evoked by oral mucosal upon thermal temperature stimuli are limited.

Electroencephalogram (EEG) is a recording of typical physiological signals of the central nervous system (Pachori and Bajaj, 2011), which can be used to detect the response of the brain to temperature sense. The EEG was chosen as a central nervous system (CNS) index due to its importance in measuring and monitoring cerebral cortical activity as well as its sensitivity to arousal fluctuation (Evans, 1993; Makeig and Inlow, 1993). Huber et al. (2006) investigated the effects of tonic heat pain on EEG rhythmic components and found that brain power in the delta band increased in most of the brain regions during tonic heat stimuli, while the theta band power in the frontotemporal diminished. In the meanwhile, the power in the alpha band was reduced, while beta power was increased (Huber et al., 2006).

Sympathetic skin response and EEG were often used as auxiliary methods to detect functional changes in autonomic and central nerves, which were helpful for the diagnosis and prediction of clinical nervous system diseases (Bach et al., 2010; Ellaway et al., 2010; Parisi et al., 2011). However, the roles played by the central nervous system as well as the autonomic nervous system (ANS) in thermal perception of the oral mucosa are unclear. Moreover, to the best of our knowledge, this was the first attempt to associate SSR with EEG to measure thermal perception of the oral mucosa, which could reflect the nerve function of ANS and CNS.

Therefore, the aims of this study were to (1) investigate and map the SSR threshold temperature in the oral mucosa at various sites in healthy young adults, (2) measure the SSR and EEG triggered in different areas of the oral mucosa under various levels of thermal stimuli, (3) explore the potential relationship between SSR and EEG, and (4) provide a theoretical basis for diagnosis and treatment of patients with abnormal temperature perception or BMS.

## Materials and Methods

### Participants

Forty healthy subjects from Tianjin Medical University were included in this study, including 24 men and 16 women, aged 19–28 years old, with a mean age of 23 ± 3 (Supplementary Table 1).

The inclusion criteria were as follows: (1) participants who had complete dentition without any mucosal diseases or abnormal temperature sensation in the oral cavity; (2) participants without the intake of psychotropics or drugs inhibiting glandular secretion in the past 2 weeks, such as diazepam and atropine; (3) participants with normal cardiac function, without any heart-related diseases; and (4) participants who did not participate in other medical experiments or clinical trials in the past 2 months. Subjects, with psychiatric or neurologic disorders, or without written informed consent, were excluded from the study. In addition, participants whose waveform could not be obtained were excluded from the final analysis.

This study was approved by the Ethics Committee of Tianjin Medical University (No. TMUhMEC20210201) and has been performed in accordance with the ethical standards laid down in the 1964 Declaration of Helsinki. Informed consent was obtained before the study. Participants were able to withdraw from this study at any time during the period of the study.

### Instrumentation of the Experiment

The thermal stimulation instrument worked on the principle of intelligent temperature control (Chinese Patent No. ZL202121087859.2) (Supplementary Figure 1). Hot temperature stimuli was imposed on the oral mucosa via a 5 mm × 5 mm Peltier semiconductor chip. Real-time data from the sensor were transmitted to the temperature control computer where fluctuations of ±0.5°C would be recorded (Supplementary Figure 2). The stability of real-time temperature was attained by a PT100 sensor (Heraeus, Shanghai, China) temperature control system.

An NDI-92 nerve/electromyogram evoked potential instrument (Shanghai Poseidon Medical Electronic Instrument Co., Ltd., Shanghai, China) was used to test SSR. It was provided by the Stomatological Anatomy and Physiology Laboratory of the Stomatological Hospital of Tianjin Medical University.

EEG signals were collected by a 32-channel Quik-Cap with a NuAmps amplifier (Compumedics Neuroscan, Australia).

### Oral Mucosa Partitions

**FIGURE 1:**
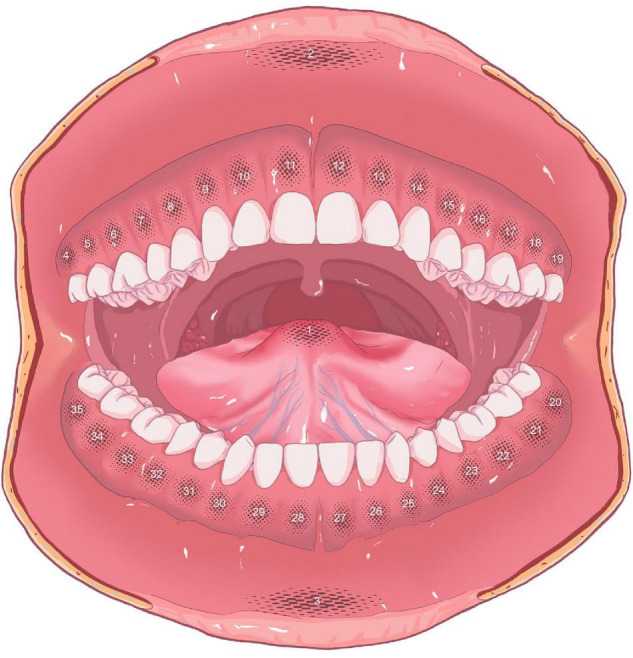
Partitions of oral mucosa (partition 1: tip of the tongue; partition 2: upper lip; partition 3: lower lip; partitions 4–35: gingival areas).

To make the results more representative and reliable, the oral mucosa in the labial area, gingival area, and anterior 2/3 of the tongue were fully measured. The authors distinguished the mucosa at the tip of the tongue and the central areas of the upper lip and lower lip as partitions 1–3, respectively. Then, according to the position of teeth, from the right maxillary third molar, the gingival mucosa was defined as area 4–35, in a clockwise direction (Figure 1).

### Electroencephalogram Electrodes of Scalp Partitions

**FIGURE 2:**
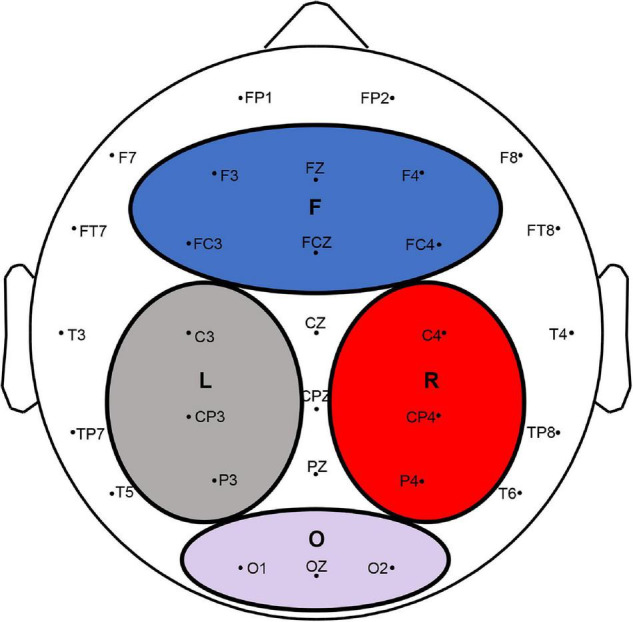
EEG electrodes and four divided regions (F, L, R, and O indicated the frontal, left parietal, right parietal, and occipital lobes, respectively).

To compute global frequency changes, electrodes were selected and grouped into four main areas: the frontal, left parietal, right parietal, and occipital areas (Zhang et al., 2016). The frontal region included F3, Fz, F4, Fc3, FCz, and Fc4. The left region included C3, Cp3, and P3, and the right region included C4, Cp4, and P4. The occipital region included O1, Oz, and O2 (Figure 2).

### Sympathetic Skin Response Threshold Temperature Measurements

Prior to the testing, participants were asked to have a rest in a supine position for 10 min, during which the procedures of testing were introduced, and participants were instructed on how to collaborate with the investigators. The testing was performed in a quiet, properly shaded room with an indoor temperature of 22–25°C between 3 and 5 pm.

Two hydrogel electrodes were attached to the palm and the back of the non-dominant hand of the subjects, whereby the palmar electrode served as the recording electrode and the dorsal electrode as the reference electrode. The grounding wire was placed on the wrist. The bandwidth of the Butterworth bandpass filter was set between 0.5 and 30 Hz, and the sampling frequency of SSR was 120 Hz. The analysis time was 10 s, during which the temperature of the palm skin was above 32°C.

First, participants were asked to gargle with water at 36°C which was the baseline temperature of the stimulus. The Peltier semiconductor chip was then placed on each area for at least 3 s. After that, the chip was removed from the testing area. If SSR could not be recorded, the temperature was set to increase by 1°C and the test was repeated. Then, the SSR threshold temperature was recorded until the SSR waveform was evoked (Supplementary Figure 3). The lowest temperature value that satisfied both of the above criteria was defined as SSR threshold temperature.

The value of SSR threshold temperature at each partition was tested twice. If the difference between these two values was significant (more than 10% of the first value), a third test would be performed. Two values that showed the smallest deviation were selected out of three results, and their mean value was defined as the final SSR threshold temperature. All partitions were tested in the same way (Supplementary Figure 4). Between two tests, the subject was required to have a short break for 1 min and gargle 36°C water for 10 s. Irregular time intervals and non-adjacent stimulation sites were used to reduce the habituation.

### Sympathetic Skin Response and Electroencephalogram Tests

Sympathetic skin response and EEG tests were conducted after the SSR threshold temperature test. Before the testing of SSR and EEG, 5-min EEG signals were recorded as a baseline EEG for each subject in a supine position with eyes closed.

Electroencephalogram electrodes were placed in the testing areas according to the extended international 10–20 system. The sampling frequency of EEG was 1,000 Hz, and the contact resistance between the electrode and scalp was less than 5 kΩ. To simultaneously perform SSR and EEG tests, a trigger device was designed and used by the researchers to synchronize the acquisition of signals. The onset of each thermal stimuli automatically excited the trigger operation, which allowed the synchronized acquisition of the SSR and EEG signals.

There were four different levels of temperature stimuli in SSR and EEG tests. A temperature of 45°C was identified as the initial temperature for the tests, and the temperature gradient was set to 3°C. Therefore, the stimulation temperatures were 45, 48, 51, and 54°C, respectively. Each testing area received four different levels of temperature stimuli.

Considering the physiological nature of the oral cavity, some representative areas were selected for the SSR and EEG tests, including (1) the central areas of incisors, premolars, and molars in maxillary and mandibular dentition, (2) the central area of the inner side of the upper and lower lip, and (3) the tip of the tongue. Since the SSR threshold temperatures were with no significant difference in premolar and molar areas, the authors, therefore, grouped these adjacent areas and irregular time intervals and non-adjacent stimulation areas were used to reduce the habituation. Each stimulation lasted for 3–5 s. Each area was tested three times, and the mean value was taken. All areas were tested in the same way.

### Subjective Numerical Pain Ratings

The participants were asked to rate their pain intensity on a visual analog scale (VAS). The scores were summed across categories and can range from 0 to 10. 0 points represented no pain; a score of 1–3 represented mild pain; a score of 4–7 represented moderate pain; and a score of 7–10 represented severe pain. The rating was obtained at the end of each recording in SSR and EEG tests.

### Analysis

#### Data Collection and Management

There were many essential parameters in the analysis of SSR, such as latency, amplitude, and waveform (Gutrecht, 1994). The latency of SSR signified the start of the record as the first deflection from the baseline. The value between the highest negative wave and the highest positive wave was known as the amplitude. In addition, the SSR area (the area under the negative component of the waveform) was also recorded (Bir et al., 2005). In this study, the SSR data were stored by the software in the electromyography (EMG) machine. The amplitude, latency, and area of SSR, in this study, were analyzed by MATLAB.

Electroencephalogram processing and analysis were also performed in MATLAB. The recorded data were bandpass-filtered (0.05–100 Hz). The line noise of 50 Hz was suppressed by a notch filter. The eye movement and muscle artifacts were corrected by independent component analysis (ICA) using EEGLAB (version 13.6.5b). Spectral analysis was performed for each data segment by the periodogram method (Akin and Kiymik, 2000). Next, the average power of the alpha and beta bands of EEG data after each temperature stimuli for all subjects for all 32 channels was calculated. The trend of the average power of the EEG delta, theta, alpha, and beta bands was analyzed and investigated according to the settings of different temperatures and thermal stimuli of different areas within the oral mucosa.

#### Statistical Analysis

Statistical analysis was performed using SPSS statistics version 25.0 (SPSS, Chicago, IL, United States). All data were tested for normality using the Shapiro–Wilk normality test. The SSR threshold temperature values were analyzed by a one-way ANOVA. The relationship between gender and SSR temperature threshold was analyzed using a generalized estimation equation (GEE), in which SSR temperature threshold was defined as the dependent variable, while partitions and gender were covariables. The SSR and EEG data were analyzed by a two-way repeated-measures ANOVA. The Bonferroni test was used when the variances were homogeneous, while Dunnett’s T3 test was used when the variances were not homogeneous. Correlational analysis was performed using Pearson’s correlations.

## Results

### Sympathetic Skin Response Threshold Temperature of the Oral Mucosa

**FIGURE 3:**
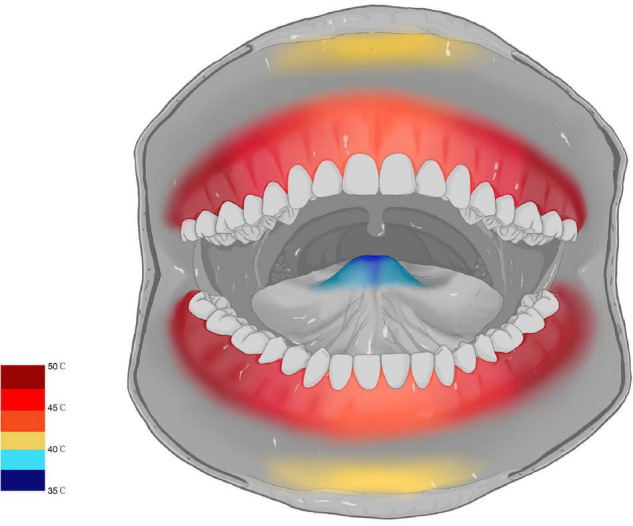
Map of the SSR threshold temperature of the oral mucosa.

The SSR waveform was successfully evoked in 35 subjects, while the other five subjects failed to evoke the SSR waveform and had no data for further analysis. In terms of oral mucosa in different areas, the SSR threshold temperatures of posterior gingival areas were higher than that in anterior gingival areas. The SSR threshold temperature of partition 6 was the highest (50.18 ± 1.71°C), and the difference between the SSR threshold temperature of partition 6 and that in other areas was significant (*P* < 0.001). In contrast, partition 1 was the most sensitive area to heat stimuli, with an SSR threshold temperature value of 36.13 ± 1.95°C, and there was a significant difference between the SSR threshold temperature of partition 1 and that in other areas (*P* < 0.001). These results reflected that the sensitivity of SSR threshold temperature of oral mucosa gradually decreased from the anterior areas to the posterior areas. However, there was no significant difference between the SSR threshold temperature of symmetrical areas on either side, such as partition 8 (46.20 ± 1.65) and partition 15 (45.88 ± 1.91) (*P* = 0.65). In addition, the sensitivity of the SSR threshold temperature of the oral mucosa showed a tendency to gradually decrease from the midline backward to both sides. The SSR threshold temperature of the oral mucosa was not affected by sex since there was no significant difference between male and female volunteers (men 45.12 ± 3.57°C; women 44.96 ± 3.43°C, OR = 1.17, *P* = 0.57). Finally, the map of the SSR threshold temperature of the oral mucosa was formed by the data obtained from the study (Figure 3).

### The Parameters of Sympathetic Skin Response

**FIGURE 4:**
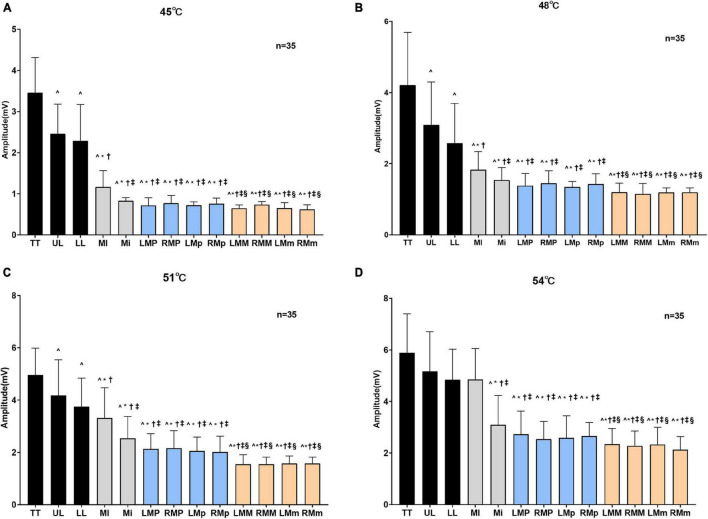
Amplitudes of SSR obtained by stimulating different areas at different degrees of temperatures. The amplitudes of SSR obtained by stimulating different areas when oral mucosa was stimulated at 45°C **(A)**, 48°C **(B)**, 51°C **(C)**, and 54°C **(D)**. TT, tip of the tongue; UL, upper lip; LL, lower lip; MI, maxillary incisor area; Mi, mandibular incisor area; LMP, left maxillary premolar area; RMP, right maxillary premolar area; LMp, left mandibular premolar area; RMp, right mandibular premolar area; LMM, left maxillary molar area; RMM, right maxillary molar area; LMm, left mandibular molar area; RMm, right mandibular molar area. ^*P* < 0.05 vs. TT, *P < 0.001 vs. UL, †*P* < 0.001 vs. LL, ^‡^*P* < 0.001 vs. MI, ^§^*P* < 0.001 vs. Mi.

A two-way repeated-measures ANOVA revealed a significant effect of area (*F* = 279.30, *P* < 0.001), temperature (*F* = 694.20, *P* < 0.001), and a significant area × temperature interaction on SSR amplitude (*F* = 5.51, *P* < 0.001) (Supplementary Table 2). At stimulation temperatures of 45, 48, and 51°C, the values of SSR amplitude in the anterior area of the oral cavity were significantly higher than those in the posterior area. In particular, the SSR amplitude value in the tip of the tongue, which was the highest value of SSR amplitude, was significantly greater than those in all other areas (*P* < 0.001), while SSR amplitudes recorded in premolar and molar areas were lower. In addition, the second and third highest values of SSR amplitude were detected in the region of the upper lip and the lower lip, respectively. In gingival areas, the first highest value of the SSR amplitude was obtained in the maxillary incisor area with significant statistical difference (*P* < 0.001). However, there was no statistical difference between the SSR amplitude obtained in the premolar and molar areas (*P* > 0.05). At the temperature of 54°C, no significant statistical difference was observed between the values of the SSR amplitude in the tip of the tongue, lips area, and the maxillary incisor area. However, compared with SSR amplitudes in the premolar or molar areas, there were significant differences between these areas (*P* < 0.001) (Figure 4). The changes in the SSR amplitudes with various areas at four different levels of stimulation temperatures followed the same trend. In addition, the SSR area showed similar trends (Table 1).

**TABLE 1:** Area of SSR obtained by stimulating different areas at different degrees of temperatures (mean ± SD).

Areas	SSR area (mVS)			
	45°C	48°C	51°C	54°C
Tip of tongue	16.10 ± 0.94	17.01 ± 1.56	19.42 ± 1.87	21.46 ± 1.70
Upper lip	13.59 ± 1.54*	14.29 ± 1.09*	17.46 ± 1.51*	18.67 ± 1.08
Lower lip	11.36 ± 1.06*	13.14 ± 1.03*	14.73 ± 1.01*	17.01 ± 0.65
Maxillary incisor area	9.13 ± 0.97*†	11.18 ± 1.24*†	13.36 ± 1.14*†	16.89 ± 1.63
Mandibular incisor area	7.23 ± 1.07*†‡	9.37 ± 1.31*†‡	12.18 ± 1.01*†‡	15.23 ± 1.17*†‡
Left maxillary premolar area	5.75 ± 0.87*†‡	6.74 ± 0.96*†‡	8.87 ± 0.97*†‡	9.45 ± 0.78*†‡
Right maxillary premolar area	5.10 ± 0.75*†‡	6.05 ± 0.72*†‡	9.63 ± 1.04*†‡	10.97 ± 1.09*†‡
Left mandibular premolar area	5.28 ± 0.80*†‡	6.27 ± 1.03*†‡	8.57 ± 1.21*†‡	10.79 ± 0.92*†‡
Right mandibular premolar area	4.79 ± 0.67*†‡	7.16 ± 0.84*†‡	9.68 ± 0.76*†‡	10.58 ± 1.06*†‡
Left maxillary molar area	2.97 ± 0.32*†‡§	4.26 ± 0.58*†‡§	5.31 ± 0.47*†‡§	6.21 ± 0.45*†‡§
Right maxillary molar area	3.12 ± 0.37*†‡§	4.02 ± 0.50*†‡§	4.70 ± 0.68*†‡§	6.36 ± 0.78*†‡§
Left mandibular molar area	3.10 ± 0.42*†‡§	3.70 ± 0.50*†‡§	5.07 ± 0.46*†‡§	6.16 ± 0.59*†‡§
Right mandibular molar area	3.33 ± 0.36*†‡§	3.94 ± 0.44*†‡§	4.95 ± 0.51*†‡§	6.38 ± 0.58*†‡§

*P < 0.001 vs. tip of the tongue.

*†P < 0.001 vs. upper lip.*

*^‡^P < 0.001 vs. lower lip.*

*^§^P < 0.001 vs. maxillary incisor area.*

There were significant differences in the SSR amplitudes obtained at different degrees of thermal stimuli (*P* < 0.001). Comparing the values of the area of SSR at different temperatures, significant differences (*P* < 0.001) were observed except at 45 and 48°C, and at 51 and 54°C. In addition, the statistical results showed that the amplitude and the area of SSR were both positively related to the intensity of temperature stimuli (Supplementary Tables 3, 4), and there was a high positive correlation between the SSR amplitude and VAS score (Supplementary Table 5).

There were significant differences in the latency of SSR between the tip of the tongue and molar areas of the oral cavity at different degrees of temperatures (*P* < 0.001) (Table 2). However, there was no significant difference in the latency of SSR between different degrees of temperatures (*P* > 0.05).

**TABLE 2:** Latency of SSR was obtained by stimulating different temperatures in different areas (Mean ± SD).

Areas	Latency (s)			
	45°C	48°C	51°C	54°C
Tip of tongue	1.41 ± 0.06	1.38 ± 0.07	1.36 ± 0.07	1.29 ± 0.06
Upper lip	1.51 ± 0.06	1.49 ± 0.06	1.47 ± 0.06	1.40 ± 0.07
Lower lip	1.56 ± 0.06	1.52 ± 0.06	1.48 ± 0.07	1.47 ± 0.03
Maxillary incisor area	1.61 ± 0.05	1.57 ± 0.04	1.55 ± 0.06	1.53 ± 0.07
Mandibular incisor area	1.67 ± 0.05	1.66 ± 0.05	1.63 ± 0.06	1.57 ± 0.06
Left maxillary premolar area	1.70 ± 0.05	1.66 ± 0.08	1.61 ± 0.07	1.60 ± 0.06
Right maxillary premolar area	1.71 ± 0.06	1.63 ± 0.07	1.60 ± 0.06	1.58 ± 0.07
Left mandibular premolar area	1.69 ± 0.07	1.62 ± 0.08	1.61 ± 0.05	1.60 ± 0.04
Right mandibular premolar area	1.68 ± 0.07	1.67 ± 0.07	1.65 ± 0.09	1.60 ± 0.06
Left maxillary molar area	1.79 ± 0.10*	1.76 ± 0.06*	1.75 ± 0.12*	1.71 ± 0.08*
Right maxillary molar area	1.80 ± 0.11*	1.78 ± 0.10*	1.72 ± 0.06*	1.67 ± 0.09*
Left mandibular molar area	1.81 ± 0.10*	1.78 ± 0.08*	1.75 ± 0.10*	1.74 ± 0.08*
Right mandibular molar area	1.81 ± 0.08*	1.79 ± 0.06*	1.76 ± 0.07*	1.73 ± 0.08*

*P < 0.001 vs. tip of the tongue.

### The Average Power of Electroencephalogram

**FIGURE 5:**
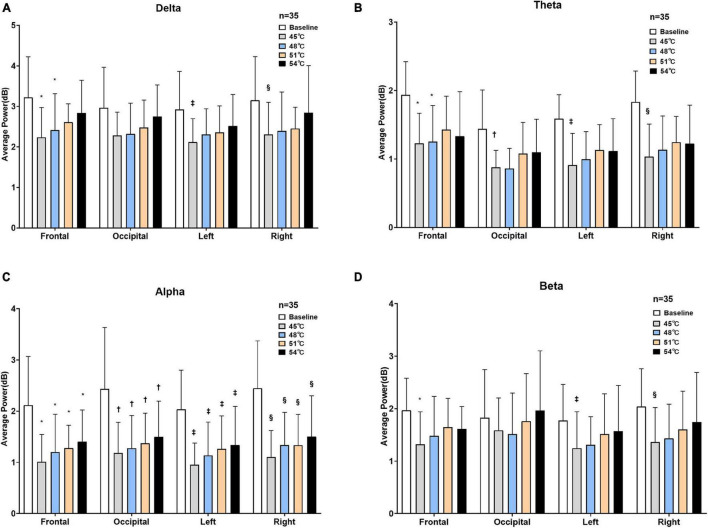
Average power of four brain areas under two states. Delta **(A)**, theta **(B)**, alpha **(C)**, and beta **(D)** power at baseline and four degrees of thermal stimulus in the frontal, occipital, left parietal, and right parietal lobes (*P < 0.001 vs. baseline in the frontal lobe, †*P* < 0.001 vs. baseline in the occipital lobe, ^‡^*P* < 0.001 vs. baseline in the left parietal lobe, ^§^*P* < 0.001 vs. baseline in the right parietal lobe).

To observe and analyze the changes in the EEG of healthy subjects in two states (rest and thermal stimuli), the average power of the baseline and four degrees of temperatures in each frequency band were calculated in each of the four areas of the brain (Figure 5). A two-way repeated-measures ANOVA revealed that temperature greatly affected the average power of delta band (*F* = 23.25, *P* < 0.001), theta band (*F* = 46.97, *P* < 0.001), alpha band (*F* = 60.28, *P* = 0.10), and beta band (*F* = 11.19, *P* = 0.02). However, neither the areas (frontal, left parietal, right parietal, and occipital areas) nor the interaction effect (area × temperature) affected the average power of all four bands (*P* > 0.05) (Supplementary Table 6). Delta band: Compared to the baseline, the delta power decreased at each degree of thermal stimuli in all four brain areas. The statistical difference was observed between baseline and 45°C (*P* = 0.01) and 48°C (*P* = 0.01) in the frontal area, while in the left and right parietal area, only between baseline and 45°C (*P* < 0.001). Theta band: Compared to the baseline, the theta power decreased at each degree of thermal stimuli in all four brain areas. In the frontal region, there were statistical differences between baseline and 45°C (*P* < 0.001) and 48°C (*P* < 0.001), while in the occipital area, and left and right parietal area, only between baseline and 45°C (*P* < 0.001). Alpha band: Compared to the baseline, the alpha power also decreased at each degree of thermal stimuli in all four brain areas. There were statistical differences between baseline and thermal stimuli at all four degrees in all four brain areas (*P* < 0.001). Beta band: Compared to the baseline, the beta power also decreased at each degree of thermal stimuli in all four brain areas except 51 and 54°C in the occipital area. Statistical difference was observed in the frontal, left, and right parietal areas between baseline and 45°C (*P* < 0.001).

**FIGURE 6:**
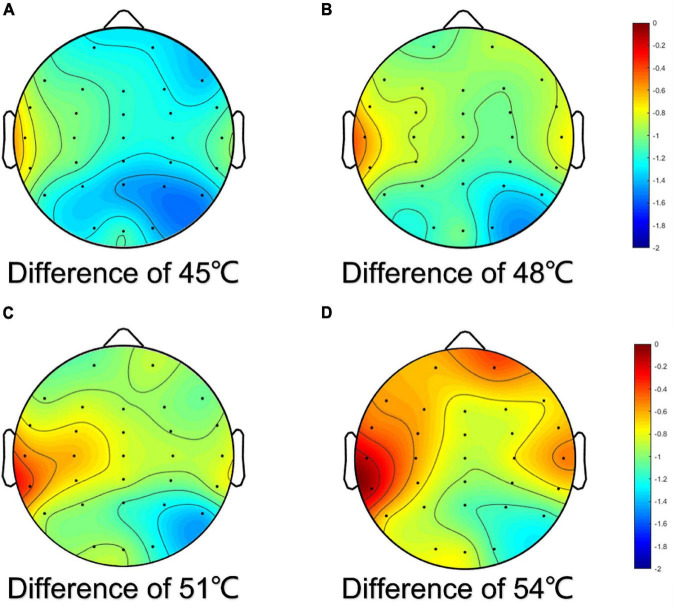
Topographic map of difference of alpha power for EEG electrodes. The difference was the power of baseline minus the power of thermal stimuli at 45°C **(A)**, 48°C **(B)**, 51°C **(C)**, and 54°C **(D)**, respectively.

The results showed that the reduction in the alpha frequency band was most obvious after thermal stimuli, and there were statistical differences between baseline and thermal stimuli in all four degrees of temperatures (*P* < 0.001) (Figure 5). To test which part of the brain areas had the most pronounced reduction at the alpha band, power change was calculated at each electrode. The results showed that the reduction in alpha power was in the global brain, and the reduction was mainly concentrated in the right parietal area near electrode P4 (Figure 6).

## Discussion

### Partitions and Sympathetic Skin Response Threshold Temperature of the Oral Mucosa

In terms of innervation, the areas of maxillary labial, buccal, and gingival mucosa were mainly innervated by the maxillary nerve, while the areas of mandibular labial, buccal, and mandibular gingival mucosa were primarily innervated by the mandibular nerve (Yamauchi et al., 2012). The anterior 2/3 area of the dorsal tongue mucosa was mainly innervated by the lingual nerve, and the latter 1/3 by the glossopharyngeal nerve—the ninth pair of the cranial nerve. In addition, Lauria et al. (2005) suggested that neurologic disturbance could act as a potential risk factor for patients with BMS. The patients with BMS were usually suffering from persistent complex pain and abnormal temperature perception. The areas we measured were more sensitive in sensing temperature compared with the other tissues of the oral cavity, such as the soft palate, and posterior tongue. Specifically, the lip and the tip of the tongue were the prominent areas of patients with BMS. This could provide a clue for further study in BMS. Based on the complicated nerve innervation and characteristic of oral mucosa disease, the fine partitions were divided by researchers. Furthermore, our results demonstrated that the SSR threshold temperature values were significantly different in different areas of the oral mucosa. The findings and ideas could generate hypotheses for further exploration of whether the patients with BMS developed autonomic neuropathy.

The differences in the sensitivity to heat among the oral partitions are caused by a series of factors, including differences in receptor location or type within the oral mucosa, diversities in innervation density, and variables related to the thermal conductivity of the oral mucosa (Green, 1984). For instance, the anterior area of the oral cavity contained more nerve endings than the posterior area, which may contribute to the superior sensitivity in the tip of the tongue area compared to the posterior gingival areas (Capra, 1995). Therefore, the sensitivity of the SSR threshold temperature of oral mucosa showed a tendency of gradual decrease backwardly bilaterally from the midline.

In addition, the degree of keratinization and physiological function of the oral mucosa were different. Compared with the lining (lip) and the specialized mucosa (tongue), the masticatory mucosa (gingiva) contacted food more frequently; therefore, its degree of keratinization was higher, leading to a higher SSR threshold. According to the findings in this study, sex had no significant effect on the SSR threshold temperature of the oral mucosa. This may relate to the small sample size, and larger samples for verification will be needed in the future. The SSR threshold temperature of oral mucosa with a high degree of keratinization was also high. Furthermore, we found that people with unilateral chewing habits had a higher tolerance to heat temperature on the preferred side than on the non-preferred side, which was more sensitive to heat. However, in this study, we observed that the SSR could not be evoked in five subjects, which did not result from the equipment, experimental process, or environmental factors, thus requiring further investigation.

### Sympathetic Skin Response Evoked by Thermal Stimuli

The tolerance of heat in different areas of oral mucosa was different. In general, the oral cavity mucosa could tolerate higher temperatures than the lip, and a temperature below 60°C will not cause harmful results. Forty-five degrees Celsius was defined as the baseline temperature in the SSR test based on the pre-experiment. In the pre-experiment, the stimulation temperature started at 36°C and increased by 1°C each time. First, it was noted that SSR began to appear frequently only after temperature stimuli of 43°C. However, SSR could not be evoked stably until each temperature stimuli was applied at approximately 45°C. Therefore, a temperature stimuli of 45°C was used to ensure that the SSR could be evoked every time and that the waveform of the SSR was stable. The authors compared the efficacy of temperature gradients of 3 and 1°C. Eventually, the gradient of 3°C was determined as ideal to test the minimum difference of SSR under different stimulation temperatures, and great differences between the amplitudes obtained at different temperatures could be observed and the areas of SSR showed the same trend. Furthermore, this study suggested that the amplitudes and the areas of SSR were positively correlated with the intensity of stimulation (Toyokura, 2003). At the same time, the SSR amplitudes and the areas obtained by the same degree of thermal temperature in different areas of the oral mucosa were different, which proved that the afferents and efferents of these areas were not innervated by the same nerve. In addition, the correlation between the SSR amplitude and VAS score was positive. Whether a direct, proportional relationship between these two parameters still needs further studies.

However, under 54°C stimulation, the amplitude of the SSR in the tip of the tongue was close to that in the upper and lower lip areas as well as the maxillary incisor area. These results further supported the idea that nociceptors could be activated at higher temperatures and subsequently initiate an extraordinary stress response (Green, 1984). Therefore, it has been hypothesized that the primary protection mechanism of the human body will be aroused when the temperature increases to a certain value. In addition to the innervation and degree of keratinization of the oral mucosa, an appropriate temperature of thermal stimuli should be taken into consideration in future research.

However, there were no significant differences in the latency of SSR between four degrees of temperatures, and the reason for differences in the latency of SSR between areas was that these areas were innervated by different nerves and the nerve conduction rate was different. Although the latency was one of the most essential factors of SSR. However, in this study, the participants were healthy young adults and the differences in latency were not obvious. Moreover, this study focused more on the relationship between SSR and EEG. Therefore, the study of latency was only a small fraction, and we will further study the difference between healthy people and patients with BMS.

### Changes in Electroencephalogram

Delta waves (0.5–4 Hz) were the slowest waves. The waveform used in the sleep assessment was the 0.5–2 Hz band. These waves were commonly found in the frontal lobe and have a relatively large amplitude of activity, normally 75 μV or even greater, with no fixed waveform duration, and were more pronounced during deep sleep or coma. Theta waves (4–8 Hz) were often found in the central region, with no fixed amplitude range, mostly in areas of the brain unrelated to hand function, and were easily detected during the shift from relaxation to sleep. Alpha waves (8–13 Hz) often appeared in the parieto-occipital region, and normal alpha waves were synchronized and symmetrical in the cerebral hemispheres and were easily detected in the state of quiet, awake, relaxed, and eyes closed resting. Beta waves (13–30 Hz) were commonly found in the frontal and central regions and were often present in states of positive thinking, concentration, anxiety, tension, and agitation. Beta waves were decreased during deep sleep, reappear during rapid-eye-movement sleep, and vary in amplitude in both hemispheres which should exceed 50% (Chokroverty, 2014).

The observed decrease in alpha power during painful stimulation was a robust finding that previous studies have also repeatedly found. Dowman et al. (2008) revealed that the lower central alpha power during thermal stimuli at the central electrodes might be relevant to pain-related activation in the primary somatosensory cortex (SI) and the somatosensory associated areas located in the parietal operculum and insula. Tension heat pain induced by heat pulses above the pain domain decreased central alpha 1 and alpha 2 activity, and the EEG pattern of lower central alpha power was related to acute human pain (Giehl et al., 2014). Zhang et al. (2016) suggested that the alpha band played a key role in invoking responses in higher hierarchical nodes of the brain such as the anterior cingulate cortex (ACC) and the ACC played an essential role in the emotional and perception aspects of pain processing. Our results showed that thermal stimuli suppressed the alpha rhythm, which was consistent with these previous findings. It has been hypothesized that as the thermal stimuli was applied, the subjects began to feel nervous and more attention was paid to uncomfortable sensations, even pain, and perhaps they acted more tensed (Chang et al., 2001; Huber et al., 2006; Herrmann et al., 2016; Jurewicz et al., 2018). Zhang et al. (2016) found a decrease in theta power in their study of the effects of tonic thermal pain on EEG rhythm, which is consistent with our study, and the change in beta power was most likely related to the presence of more noise in this frequency band (Giehl et al., 2014). However, some other studies in tonic pain stimulation have found that delta power showed an upward trend which was inconsistent with our finding (Ferracuti et al., 1994; Chang et al., 2002; Huber et al., 2006). This may be because, in previous studies, the thermal stimuli was mostly applied to the outer epidermis such as hands or legs, while our stimulation was applied to the oral mucosa, and there were differences in nerve conduction mechanism and receptors between the skin and oral mucosa (Capra, 1995).

Our results showed that the decreasing trend of alpha power in all four brain areas became less obvious as the temperature of thermal stimuli increased. There was no doubt that the psychological state of the subjects at rest was relaxed, as indicated by the maximum alpha power at baseline. However, there were only statistical differences between baseline and certain temperature stimuli, and no statistical differences between temperatures. In addition, previous studies often used much larger differences between thermal stimulus intensities, and the stimulation sites were different from our study (Nickel et al., 2017; Albu and Meagher, 2019). Therefore, different experimental paradigms may result in different results. The stimulation time in our study was shorter than that in continuous stimulation. There were many reasons for the differences between studies such as stimulation protocol, stimulus intensity, and electrode locations (Tripanpitak et al., 2021). In future research, we will make full use of the 32-channel data collected from the perspective of the brain network to analyze the information flow between different nodes in detail, including the characteristics of the brain network, such as the flow gain, clustering coefficient, and characteristic path length, to investigate what difference between different temperature and the EEG brain network connection to thermal stimuli (Bunk et al., 2018).

Physiological studies have confirmed that during the transmission of human oral temperature stimuli from the periphery to the center, the oral cavity received oral internal temperature and cold sensory stimuli through mucosal receptors and converged to the caudate nucleus of the bilateral trigeminal spinal tract nucleus through the tympanic cord nerve fibers, trigeminal nerve, and lingual nerve fibers. Then, the caudate nucleus of the spinal trigeminal nucleus further transmitted the warm pain information to the ventroposteromedial nucleus and ventroposterolateral nucleus of the thalamus (Adams, 1976) and then transmitted the information to the cerebral cortex after changing neurons (Darian-Smith et al., 1979; Kawahara et al., 1986). Temperature and pain afferent down to the spinal nucleus of the trigeminal nerve, and the ascending conduction pathway of pain and temperature sensation came from the spinal nucleus of the trigeminal nerve. Bromm (2001) reported that pain response in the brain was a complex process which involved multiple cortical brain areas such as the primary and secondary somatosensory cortices, the insular cortex (IC), and the ACC. The temperature receptors in the cerebral cortex were located in the paracentral part of the parietal cortex of the posterior central gyrus, and these areas were referred to as the somatosensory region I which was located in the left and right parietal areas of the scalp electrode (Brownsett and Wise, 2010). Our results revealed that there was a reduction in alpha power in both the left and right parietal areas, with the greatest change in the right parietal areas and contralateral somatosensory regions (SI and SII) near the P4 electrode were most responsive to sensing and relaying pain caused by thermal stimuli. This was the same as the consensus that SI and SII are part of the pain network (Oertel et al., 2012).

### The Relationship Between Sympathetic Skin Response and Electroencephalogram

Lim et al. (1996) reported a strong correlation between beta-band activity and skin conductance level, due to these measures were shared reticulo-thalamo-hypothalamo-cortical networks; therefore, a functional link between these measures might be predicted. Belyavin and Wright (1987) reported that beta activity can serve as a good discriminator for worsening the vigilance level. Garcia et al. (2011) suggested an electrophysiological framework with interactions between brain dynamics and autonomic responses elicited by emotional engagement in a working memory task. In addition, the sympathetic changes in the skin conductance level have been related to mental stress and emotion regulation (Crone et al., 2004; Werner et al., 2009). As the temperature stimuli was increased, in this study, the amplitudes and the area of SSR which could represent the intensity of perception of the oral cavity were both gradually increased, below the noxious temperature level. Meanwhile, the average powers of the alpha band that could represent relaxation in the brain cortex also showed an upward trend. According to the results of SSR and EEG, this phenomenon suggested that the oral mucosa was always sensitive to temperature stimuli and could accurately distinguish the difference in stimulation temperature. At the same time, the CNS gradually showed certain habituation as the temperature increased. The oral mucosa, especially the lips and tongue areas at the forefront, could feel the temperature of the food ingested accurately and could determine the appropriate intake time, speed, and capacity of food to avoid damage from temperature. Although the CNS has an adaptive mechanism to temperature stimuli, the oral mucosa could always maintain an accurate perception of temperature stimuli, which was beneficial to avoid injury from ingesting overheated food. However, when this adaptive mechanism upregulated the intake temperature, continuous higher temperature stimuli may damage the oral mucosa, which could cause ulcers and even induce diseases such as esophageal cancer.

These findings, including ours, bear testimony to the fact that the CNS and the ANS are structurally linked. Kenchadze et al. (2011) reported improved BMS patients’ condition using the EEG–feedback method; the clinical manifestations of some cases were evened out completely. In the meantime, in our clinical experience, patients who suffered from BMS could benefit from neurotropic medications improving the state of the autonomic nervous system. In the follow-up study, we will use the perspective of the brain network to deeply explore the response of the central nervous system to oral thermal stimuli at different temperatures and further reveal the relationship between the CNS and the ANS.

### Limitations

The degree of keratinization of the oral mucosa was related to many influencing factors such as age, dietary type, and chewing habits. There were certain differences in the degree of keratinization of the oral mucosa among people of different ages (Abu Eid et al., 2012). In addition, the distribution density and sensitivity of the receptors and nerve conduction velocity also varied among different participants. It was true that a sense of hypothetically suitable ambient temperature is subjective. It could be, therefore, dramatically various among individuals with potential influence on the tension of the nervous system. SSR was normally present in both palms and soles under the age of 60 years, but in subjects aged 60 and over, it was found in only 50% of feet and 73% of hands (Drory and Korczyn, 1993). In this study, participants were all healthy young adults whose age and level of physiological function were similar, so the data of this experiment did not reflect the trend in the change among the whole population with age. Therefore, middle-aged and older people were necessary for follow-up research.

Sympathetic skin response can be used to assess the activity of the sympathetic nervous system. However, the biggest limitation was the habituation of this procedure. The essence of habituation was unclear. For SSR, with the increase in the number of stimuli at the same stimulation site, the amplitude decreased in a short period and could recover soon (Cariga et al., 2001). The habituation of SSR was difficult to avoid. Therefore, in this study, we used irregular time intervals and non-adjacent stimulation sites to reduce habituation, although the habituation evoked by temperature stimuli was less than that evoked by electrical stimulation. However, as a definite physiological characteristic, this trend still existed. SSR characteristics depended highly on habituation. In particular, the excitability level and the surprise effect of sympathetic neurons are important factors influencing the progressive and irregular variability of the reactions during long-term experimental assessments. Reducing the influence of SSR habituation will be a focus in future research.

## Conclusion

The SSR threshold temperature of the oral mucosa varied greatly in different areas. The anterior area of the oral mucosa was the most sensitive to temperature, while the posterior area of the oral mucosa was less sensitive, which was consistent with the pattern of food intaking function of the oral cavity. The topographic map of the oral mucosa SSR threshold temperature summarily elucidated the physiological characteristics of the thermal perception pattern as well as the mechanisms of thermal habituation and protection from harmful stimuli. The action potential of SSR evoked by thermal stimuli was an objective parameter reflecting the activity of the autonomic nervous system; in this study, we hypothesized that thermal stimuli aroused SSR existed extensively over the human body than limited to the oral cavity.

Our study tried to transform the subjective perception of oral mucosa to thermal stimuli into a relatively objective and accurate quantitative description, which was helpful for us to have a deeper understanding of the neurophysiological mechanism of temperature perception of oral mucosa from a new point of view. In this study, through the perception of thermal temperature in oral mucosa, we further explored the activities, mechanisms, and influencing factors of the ANS and CNS, to better understand and diagnose BMS.

Measurement of synchronous activities of the CNS by EEG average power level indicated that both the autonomic and central nervous system played important roles in thermal perception. Further application of multiple channels of electrophysiological parameter analysis could be helpful to better understand the interrelationship between the CNS and ANS.

## Data Availability Statement

The original contributions presented in this study are included in the article/Supplementary Material, further inquiries can be directed to the corresponding authors.

## Ethics Statement

The studies involving human participants were reviewed and approved by the Ethics Committee of Tianjin Medical University (No. TMUhMEC20210201) and have been performed in accordance with the ethical standards laid down in the 1964 Declaration of Helsinki. The patients/participants provided their written informed consent to participate in this study.

## Author Contributions

HZ was responsible for study conception and design, device design, and assemble. SH was responsible for volunteer recruitment, data analyses, and drafted the manuscript. ZW contributed to the data interpretation. XL contributed to the data interpretation and critical revision of the manuscript. GC and SW contributed to the study conception and design, data interpretation, critically reviewing the manuscript, and crucial guidance in the study. All authors have given final approval and agreed to be responsible for all aspects of this study to ensure accuracy and integrity.

## Conflict of Interest

The authors declare that the research was conducted in the absence of any commercial or financial relationships that could be construed as a potential conflict of interest.

## Publisher’s Note

All claims expressed in this article are solely those of the authors and do not necessarily represent those of their affiliated organizations, or those of the publisher, the editors and the reviewers. Any product that may be evaluated in this article, or claim that may be made by its manufacturer, is not guaranteed or endorsed by the publisher.

## References

[B1] Abu Eid R. Sawair F. Landini G. Saku T. (2012). Age and the architecture of oral mucosa. *Age* 34 651–658. 10.1007/s11357-011-9261-1 21559867 PMC3337934

[B2] Adams D. (1976). Keratinization of the oral epithelium. *Ann. R. Coll. Surg. Engl.* 58 351–358. 788618 PMC2491838

[B3] Akin M. Kiymik M. K. (2000). Application of periodogram and AR spectral analysis to EEG signals. *J. Med. Syst.* 24 247–256. 10.1023/a:1005553931564 11057403

[B4] Albu S. Meagher M. W. (2019). Divergent effects of conditioned pain modulation on subjective pain and nociceptive-related brain activity. *Exp. Brain Res.* 237 1735–1744. 10.1007/s00221-019-05545-8 31030281

[B5] Bach D. R. Friston K. J. Dolan R. J. (2010). Analytic measures for quantification of arousal from spontaneous skin conductance fluctuations. *Int. J. Psychophysiol.* 76 52–55. 10.1016/j.ijpsycho.2010.01.011 20144665 PMC2877802

[B6] Belyavin A. Wright N. A. (1987). Changes in electrical activity of the brain with vigilance. *Electroencephalogr. Clin. Neurophysiol.* 66 137–144. 10.1016/0013-4694(87)90183-0 2431878

[B7] Bir L. S. Ozkurt S. Daloğlu G. Kurt T. (2005). Impaired sympathetic skin response in chronic obstructive pulmonary disease. *Tohoku J. Exp. Med.* 207 243–248. 10.1620/tjem.207.243 16272793

[B8] Bromm B. (2001). Brain images of pain. *News Physiol. Sci.* 16 244–249. 11572930 10.1152/physiologyonline.2001.16.5.244

[B9] Brownsett S. L. Wise R. J. (2010). The contribution of the parietal lobes to speaking and writing. *Cereb. Cortex* 20 517–523. 10.1093/cercor/bhp120 19531538 PMC2820696

[B10] Bunk S. F. Lautenbacher S. Rüsseler J. Müller K. Schultz J. Kunz M. (2018). Does EEG activity during painful stimulation mirror more closely the noxious stimulus intensity or the subjective pain sensation? *Somatosens. Mot. Res.* 35 192–198. 10.1080/08990220.2018.1521790 30461318

[B11] Capra N. F. (1995). Mechanisms of oral sensation. *Dysphagia* 10 235–247. 10.1007/bf00431416 7493504

[B12] Cariga P. Catley M. Mathias C. J. Ellaway P. H. (2001). Characteristics of habituation of the sympathetic skin response to repeated electrical stimuli in man. *Clin. Neurophysiol.* 112 1875–1880. 10.1016/s1388-2457(01)00647-2 11595146

[B13] Chang P. F. Arendt-Nielsen L. Chen A. C. (2002). Dynamic changes and spatial correlation of EEG activities during cold pressor test in man. *Brain Res. Bull.* 57 667–675. 10.1016/s0361-9230(01)00763-8 11927371

[B14] Chang P. F. Arendt-Nielsen L. Graven-Nielsen T. Svensson P. Chen A. C. (2001). Topographic effects of tonic cutaneous nociceptive stimulation on human electroencephalograph. *Neurosci. Lett.* 305 49–52. 10.1016/s0304-3940(01)01802-x 11356305

[B15] Chokroverty S. (2014). *Atlas of Sleep Medicine* 2nd Edn. Philadelphia, PA: Elsevier/Saunders.

[B16] Crone E. A. Somsen R. J. Van Beek B. Van Der Molen M. W. (2004). Heart rate and skin conductance analysis of antecendents and consequences of decision making. *Psychophysiology* 41 531–540. 10.1111/j.1469-8986.2004.00197.x 15189476

[B17] Darian-Smith I. Johnson K. O. Lamotte C. Shigenaga Y. Kenins P. Champness P. (1979). Warm fibers innervating palmar and digital skin of the monkey: responses to thermal stimuli. *J. Neurophysiol.* 42 1297–1315. 10.1152/jn.1979.42.5.1297 114608

[B18] Dowman R. Rissacher D. Schuckers S. (2008). EEG indices of tonic pain-related activity in the somatosensory cortices. *Clin. Neurophysiol.* 119 1201–1212. 10.1016/j.clinph.2008.01.019 18337168 PMC2676940

[B19] Drory V. E. Korczyn A. D. (1993). Sympathetic skin response: age effect. *Neurology* 43 1818–1820. 10.1212/wnl.43.9.1818 8414038

[B20] Ellaway P. H. Kuppuswamy A. Nicotra A. Mathias C. J. (2010). Sweat production and the sympathetic skin response: improving the clinical assessment of autonomic function. *Auton. Neurosci.* 155 109–114. 10.1016/j.autneu.2010.01.008 20129828

[B21] Evans B. M. (1993). Cyclical activity in non-rapid eye movement sleep: a proposed arousal inhibitory mechanism. *Electroencephalogr. Clin. Neurophysiol.* 86 123–131. 10.1016/0013-4694(93)90084-9 7681379

[B22] Ferracuti S. Seri S. Mattia D. Cruccu G. (1994). Quantitative EEG modifications during the cold water pressor test: hemispheric and hand differences. *Int. J. Psychophysiol.* 17 261–268. 10.1016/0167-8760(94)90068-x 7806469

[B23] Forssell H. Jääskeläinen S. Tenovuo O. Hinkka S. (2002). Sensory dysfunction in burning mouth syndrome. *Pain* 99 41–47. 10.1016/s0304-3959(02)00052-0 12237182

[B24] Garcia A. Uribe C. E. Tavares M. C. Tomaz C. (2011). EEG and autonomic responses during performance of matching and non-matching to sample working memory tasks with emotional content. *Front. Behav. Neurosci.* 5:82. 10.3389/fnbeh.2011.00082 22203795 PMC3243924

[B25] Giehl J. Meyer-Brandis G. Kunz M. Lautenbacher S. (2014). Responses to tonic heat pain in the ongoing EEG under conditions of controlled attention. *Somatosens. Mot. Res.* 31 40–48. 10.3109/08990220.2013.837045 24320554

[B26] Green B. G. (1984). Thermal perception on lingual and labial skin. *Percept. Psychophys.* 36 209–220. 10.3758/bf03206361 6522212

[B27] Gutrecht J. A. (1994). Sympathetic skin response. *J. Clin. Neurophysiol.* 11 519–524. 10.1097/00004691-199409000-00006 7844242

[B28] Hagelberg N. Forssell H. Rinne J. O. Scheinin H. Taiminen T. Aalto S. (2003). Striatal dopamine D1 and D2 receptors in burning mouth syndrome. *Pain* 101 149–154. 10.1016/s0304-3959(02)00323-8 12507709

[B29] Herrmann C. S. Strüber D. Helfrich R. F. Engel A. K. (2016). EEG oscillations: from correlation to causality. *Int. J. Psychophysiol.* 103 12–21. 10.1016/j.ijpsycho.2015.02.003 25659527

[B30] Hilz M. J. Azelrod F. B. Schweibold G. Kolodny E. H. (1999). Sympathetic skin response following thermal, electrical, acoustic, and inspiratory gasp stimulation in familial dysautonomia patients and healthy persons. *Clin. Auton. Res.* 9 165–177. 10.1007/bf02330480 10574280

[B31] Huber M. T. Bartling J. Pachur D. Woikowsky-Biedau S. Lautenbacher S. (2006). EEG responses to tonic heat pain. *Exp. Brain Res.* 173 14–24. 10.1007/s00221-006-0366-1 16552561

[B32] Jurewicz K. Paluch K. Kublik E. Rogala J. Mikicin M. Wróbel A. (2018). EEG-neurofeedback training of beta band (12-22Hz) affects alpha and beta frequencies - A controlled study of a healthy population. *Neuropsychologia* 108 13–24. 10.1016/j.neuropsychologia.2017.11.021 29162459

[B33] Karl H. Sato A. Schmidt R. F. (1975). Electrodermal reflexes induced by activity in somatic afferent fibers. *Brain Res.* 87 145–150. 10.1016/0006-8993(75)90410-2 1125765

[B34] Kawahara K. Sawada Y. Aoki M. (1986). Dual pathways for thermal afferents from the cat’s tongue. *Brain Res.* 378 61–68. 10.1016/0006-8993(86)90286-6 3742204

[B35] Kenchadze R. Iverieli M. Okribelashvili N. Geladze N. Khachapuridze N. (2011). The psychological aspects of burning mouth syndrome. *Georgian Med. News* 194 24–28. 21685517

[B36] Koszewicz M. Mendak M. Konopka T. Koziorowska-Gawron E. Budrewicz S. (2012). The characteristics of autonomic nervous system disorders in burning mouth syndrome and Parkinson disease. *J. Orofac. Pain* 26 315–320. 23110271

[B37] Lauria G. Majorana A. Borgna M. Lombardi R. Penza P. Padovani A. (2005). Trigeminal small-fiber sensory neuropathy causes burning mouth syndrome. *Pain* 115 332–337. 10.1016/j.pain.2005.03.028 15911160

[B38] Lim C. L. Barry R. J. Gordon E. Sawant A. Rennie C. Yiannikas C. (1996). The relationship between quantified EEG and skin conductance level. *Int. J. Psychophysiol.* 21 151–162. 10.1016/0167-8760(95)00049-6 8792203

[B39] Liu H. Zheng Y. F. Li C. Y. Zheng Y. Y. Wang D. Q. Wu Z. (2015). Discovery of anti-inflammatory ingredients in Chinese herbal formula kouyanqing granule based on relevance analysis between chemical characters and biological effects. *Sci. Rep.* 5:18080. 10.1038/srep18080 26657159 PMC4674803

[B40] Makeig S. Inlow M. (1993). Lapses in alertness: coherence of fluctuations in performance and EEG spectrum. *Electroencephalogr. Clin. Neurophysiol.* 86 23–35. 10.1016/0013-4694(93)90064-3 7678388

[B41] Nakamura Y. Une Y. Miyano K. Abe H. Hisaoka K. Morioka N. (2012). Activation of transient receptor potential ankyrin 1 evokes nociception through substance P release from primary sensory neurons. *J. Neurochem.* 120 1036–1047. 10.1111/j.1471-4159.2011.07628.x 22182301

[B42] Nickel M. M. May E. S. Tiemann L. Postorino M. Ta Dinh S. Ploner M. (2017). Autonomic responses to tonic pain are more closely related to stimulus intensity than to pain intensity. *Pain* 158 2129–2136. 10.1097/j.pain.0000000000001010 28700538

[B43] Oertel B. G. Preibisch C. Martin T. Walter C. Gamer M. Deichmann R. (2012). Separating brain processing of pain from that of stimulus intensity. *Hum. Brain Mapp.* 33 883–894. 10.1002/hbm.21256 21681856 PMC6869957

[B44] Pachori R. B. Bajaj V. (2011). Analysis of normal and epileptic seizure EEG signals using empirical mode decomposition. *Comput. Methods Programs Biomed.* 104 373–381. 10.1016/j.cmpb.2011.03.009 21529981

[B45] Parisi P. Verrotti A. Paolino M. C. Castaldo R. Ianniello F. Ferretti A. (2011). “Electro-clinical syndromes” with onset in paediatric age: the highlights of the clinical-EEG, genetic and therapeutic advances. *Ital. J. Pediatr.* 37:58. 10.1186/1824-7288-37-58 22182677 PMC3267655

[B46] Petrus M. Peier A. M. Bandell M. Hwang S. W. Huynh T. Olney N. (2007). A role of TRPA1 in mechanical hyperalgesia is revealed by pharmacological inhibition. *Mol. Pain* 3:40. 10.1186/1744-8069-3-40 18086313 PMC2222610

[B47] Toyokura M. (2003). Influence of stimulus intensity on waveform of sympathetic skin response evoked by magnetic stimulation. *Clin. Neurophysiol.* 114 1423–1430. 10.1016/s1388-2457(03)00162-7 12888024

[B48] Tripanpitak K. He S. Y. Sonmezisik I. Morant T. Huang S. Y. Yu W. W. (2021). Granger causality-based pain classification using EEG evoked by electrical stimulation targeting nociceptive A delta and C Fibers. *IEEE Access* 9 10089–10106. 10.1109/access.2021.3050302

[B49] Vetrugno R. Liguori R. Cortelli P. Montagna P. (2003). Sympathetic skin response: basic mechanisms and clinical applications. *Clin. Auton. Res.* 13 256–270. 10.1007/s10286-003-0107-5 12955550

[B50] Wei H. Koivisto A. Saarnilehto M. Chapman H. Kuokkanen K. Hao B. (2011). Spinal transient receptor potential ankyrin 1 channel contributes to central pain hypersensitivity in various pathophysiological conditions in the rat. *Pain* 152 582–591. 10.1016/j.pain.2010.11.031 21211906

[B51] Werner N. S. Duschek S. Schandry R. (2009). Relationships between affective states and decision-making. *Int. J. Psychophysiol.* 74 259–265. 10.1016/j.ijpsycho.2009.09.010 19808059

[B52] Yamauchi K. Takahashi T. Kaneuji T. Nogami S. Yamamoto N. Miyamoto I. (2012). Risk factors for neurosensory disturbance after bilateral sagittal split osteotomy based on position of mandibular canal and morphology of mandibular angle. *J. Oral Maxillofac. Surg.* 70 401–406. 10.1016/j.joms.2011.01.040 21549489

[B53] Zhang C. H. Sohrabpour A. Lu Y. He B. (2016). Spectral and spatial changes of brain rhythmic activity in response to the sustained thermal pain stimulation. *Hum. Brain Mapp.* 37 2976–2991. 10.1002/hbm.23220 27167709 PMC4945371

