# Supplementary material for: A Temporospatial Study of Sympathetic Skin Response and Electroencephalogram in Oral Mucosa Thermal Perception
Source: Front Neurosci. 2022 Jul 15;16:907658. doi: 10.3389/fnins.2022.907658 (PMC9337692; doi:10.3389/fnins.2022.907658)
Supplement: Supplementary file 1 [file Data_Sheet_1.docx]

Supplementary Material


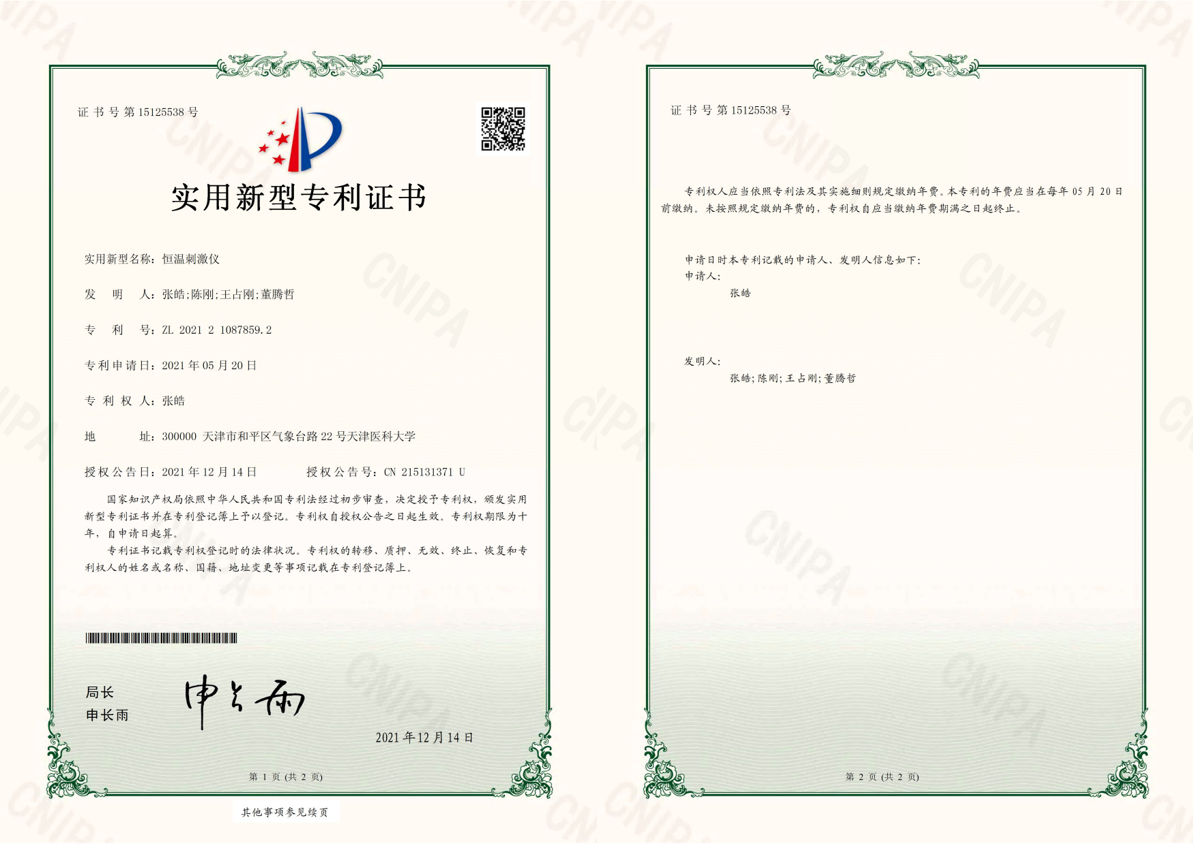


Supplementary Figure 1 The national patent certificate of constant temperature stimulator (Chinese Patent No. ZL202121087859.2).


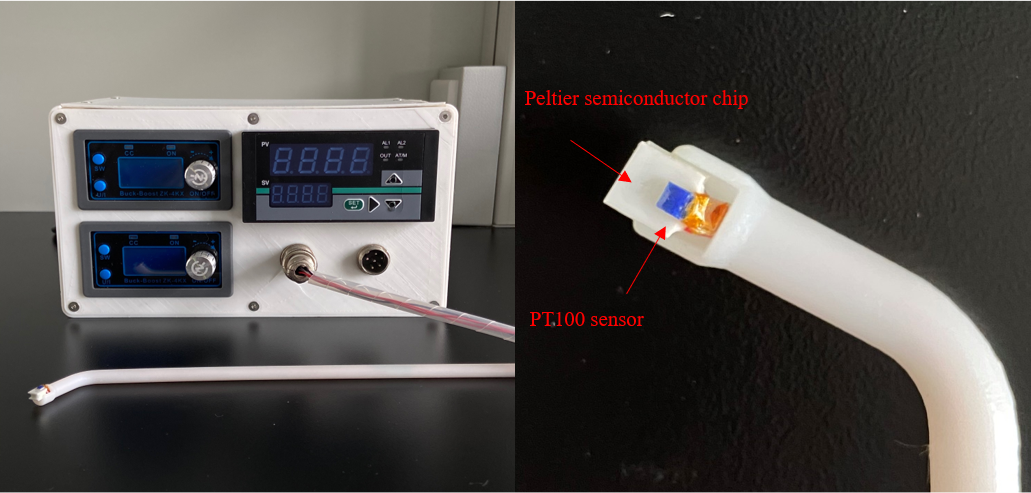


Supplementary Figure 2 The appearance of the constant temperature stimulator **(A)** and the detail of probe **(B)**.


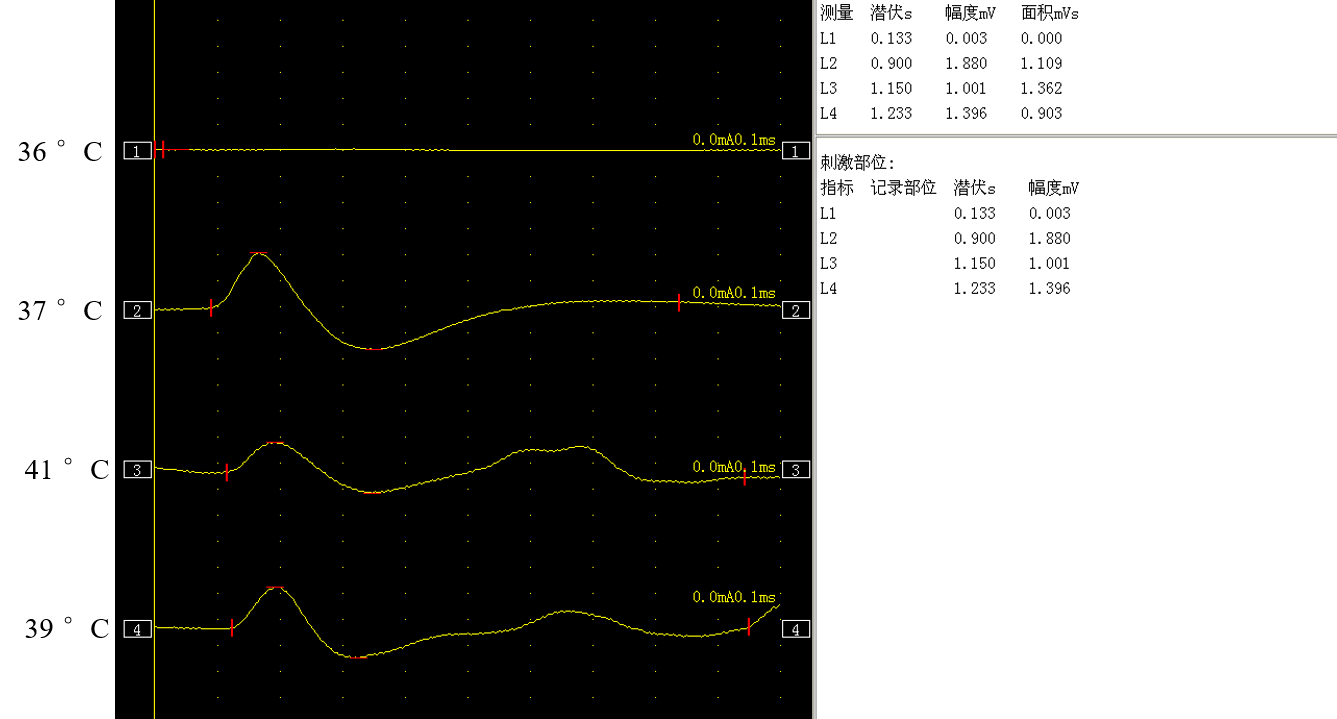


Supplementary Figure 3 The SSR waveforms of partition 1 from a subject.


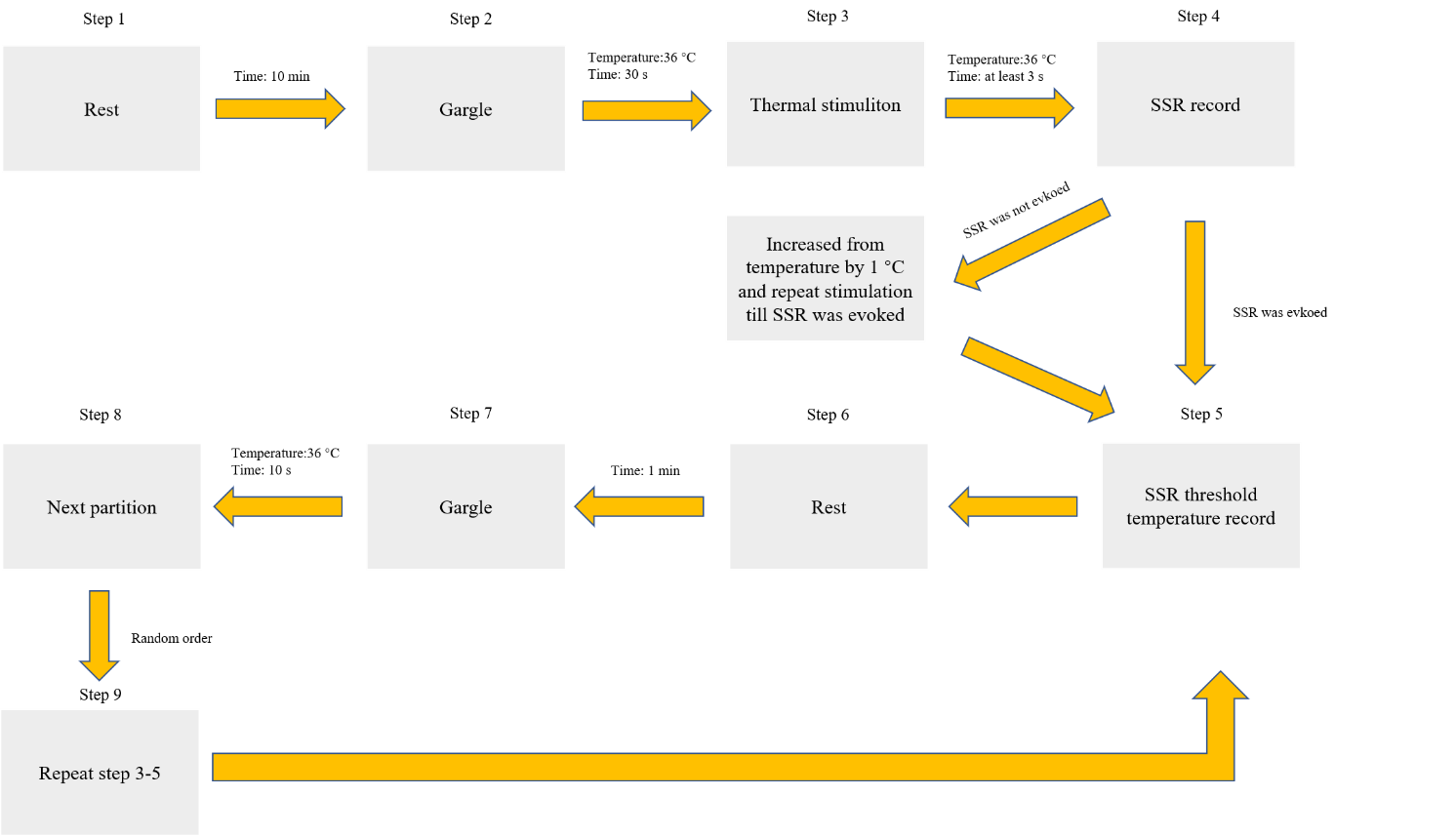


Supplementary Figure 4 The process of SSR threshold temperature measurement.

Supplementary Table 1 The characteristics of subjects.

| Subject characteristics | |
| --- | --- |
| Age | 23 ± 3 |
| Gender (Male/female) | 24/16 |
| Height (m) | 1.71 ± 0.06 |
| Mass (kg) | 58.34 ± 8.27 |
| Mean skin temperature of hand (°C) | 33.09 ± 1.02 |
| Edinburgh Handedness (Left/Right) | 5/35 |
| Heart rate (bpm) | 76.58 ± 2.41 |

Supplementary Table 2 Statistical results of the effects of area, temperature, and area × temperature interactions on the SSR amplitude.

| Factor | *F* | *P* |
| --- | --- | --- |
| Area | 279.30 | < 0.001 |
| Temperature | 694.20 | < 0.001 |
| Area × temperature | 5.51 | < 0.001 |

Supplementary Table 3 Pearson correlation analysis of SSR amplitudes in different oral areas associated with temperature.

| SSR amplitude | Temperature | |
| --- | --- | --- |
|  | *r* | *P* |
| Tip of tongue | .59 | < 0.001 |
| Upper lip | .64 | < 0.001 |
| Lower lip | .67 | < 0.001 |
| Maxillary incisor area | .84 | < 0.001 |
| Mandibular incisor area | .77 | < 0.001 |
| Left maxillary premolar area | .80 | < 0.001 |
| Right maxillary premolar area | .79 | < 0.001 |
| Left mandibular premolar area | .81 | < 0.001 |
| Right mandibular premolar area | .84 | < 0.001 |
| Left maxillary molar area | .84 | < 0.001 |
| Right maxillary molar area | .84 | < 0.001 |
| Left mandibular molar area | .85 | < 0.001 |
| Right mandibular molar area | .88 | < 0.001 |

Supplementary Table 4 Pearson correlation analysis of the SSR areas in different oral areas associated with temperature.

| SSR area | Temperature | |
| --- | --- | --- |
|  | *r* | *P* |
| Tip of tongue | .32 | .01 |
| Upper lip | .36 | .01 |
| Lower lip | .48 | <0.001 |
| Maxillary incisor area | .49 | < 0.001 |
| Mandibular incisor area | .55 | < 0.001 |
| Left maxillary premolar area | .38 | < 0.001 |
| Right maxillary premolar area | .54 | < 0.001 |
| Left mandibular premolar area | .46 | < 0.001 |
| Right mandibular premolar area | .55 | < 0.001 |
| Left maxillary molar area | .54 | < 0.001 |
| Right maxillary molar area | .44 | < 0.001 |
| Left mandibular molar area | .51 | < 0.001 |
| Right mandibular molar area | .51 | < 0.001 |

Supplementary Table 5 Pearson correlation analysis of the SSR amplitudes in different oral areas associated with VAS scores.

| SSR amplitude | VAS | |
| --- | --- | --- |
|  | *r* | *P* |
| Tip of tongue | .86 | .01 |
| Upper lip | .31 | .01 |
| Lower lip | .84 | < 0.001 |
| Maxillary incisor area | .90 | < 0.001 |
| Mandibular incisor area | .88 | < 0.001 |
| Left maxillary premolar area | .87 | < 0.001 |
| Right maxillary premolar area | .79 | < 0.001 |
| Left mandibular premolar area | .86 | < 0.001 |
| Right mandibular premolar area | .85 | < 0.001 |
| Left maxillary molar area | .81 | < 0.001 |
| Right maxillary molar area | .87 | < 0.001 |
| Left mandibular molar area | .81 | < 0.001 |
| Right mandibular molar area | .79 | < 0.001 |

Supplementary Table 6 Statistical results of the effects of band, temperature, and band × temperature interactions on the EEG average power.

| Band | Factor | *F* | *P* |
| --- | --- | --- | --- |
| Delta | Area | 2.51 | 0.06 |
|  | Temperature | 23.25 | <0.001 |
|  | Area × temperature | 0.24 | 0.99 |
| Theta | Area | 21.23 | 0.08 |
|  | Temperature | 46.97 | < 0.001 |
|  | Area × temperature | 0.44 | 0.95 |
| Alpha | Area | 3.77 | 0.10 |
|  | Temperature | 60.28 | < 0.001 |
|  | Area × temperature | 0.32 | 0.99 |
| Beta | Area | 3.30 | 0.25 |
|  | Temperature | 11.19 | 0.02 |
|  | Area × temperature | 0.54 | 0.89 |
